# Transient Expression of Hemagglutinin Antigen from Low Pathogenic Avian Influenza A (H7N7) in *Nicotiana benthamiana*

**DOI:** 10.1371/journal.pone.0033010

**Published:** 2012-03-19

**Authors:** Selvaraju Kanagarajan, Conny Tolf, Anneli Lundgren, Jonas Waldenström, Peter E. Brodelius

**Affiliations:** 1 Section for Biomaterials and Medicinal Chemistry, School of Natural Sciences, Linnaeus University, Kalmar, Sweden; 2 Section for Zoonotic Ecology and Epidemiology, School of Natural Sciences, Linnaeus University, Kalmar, Sweden; University of Waikato, New Zealand

## Abstract

The influenza A virus is of global concern for the poultry industry, especially the H5 and H7 subtypes as they have the potential to become highly pathogenic for poultry. In this study, the hemagglutinin (HA) of a low pathogenic avian influenza virus of the H7N7 subtype isolated from a Swedish mallard *Anas platyrhynchos* was sequenced, characterized and transiently expressed in *Nicotiana benthamiana*. Recently, plant expression systems have gained interest as an alternative for the production of vaccine antigens. To examine the possibility of expressing the HA protein in *N. benthamiana*, a cDNA fragment encoding the *HA* gene was synthesized *de novo*, modified with a Kozak sequence, a PR1a signal peptide, a C-terminal hexahistidine (6×His) tag, and an endoplasmic retention signal (SEKDEL). The construct was cloned into a Cowpea mosaic virus (CPMV)-based vector (pEAQ-*HT*) and the resulting pEAQ-*HT*-HA plasmid, along with a vector (pJL3:p19) containing the viral gene-silencing suppressor *p19* from Tomato bushy stunt virus, was agro-infiltrated into *N. benthamiana*. The highest gene expression of recombinant plant-produced, uncleaved HA (rHA0), as measured by quantitative real-time PCR was detected at 6 days post infiltration (dpi). Guided by the gene expression profile, rHA0 protein was extracted at 6 dpi and subsequently purified utilizing the 6×His tag and immobilized metal ion adsorption chromatography. The yield was 0.2 g purified protein per kg fresh weight of leaves. Further molecular characterizations showed that the purified rHA0 protein was N-glycosylated and its identity confirmed by liquid chromatography-tandem mass spectrometry. In addition, the purified rHA0 exhibited hemagglutination and hemagglutination inhibition activity indicating that the rHA0 shares structural and functional properties with native HA protein of H7 influenza virus. Our results indicate that rHA0 maintained its native antigenicity and specificity, providing a good source of vaccine antigen to induce immune response in poultry species.

## Introduction

Avian influenza viruses (AIV) belong to the family of *Orthomyxoviridae*, a family consisting of five different genera including the *Influenzavirus A* genus. In nature, waterfowl and shorebirds are thought to constitute the primary reservoir of influenza A virus. Virus subtypes are identified based on nucleotide sequence diversity and antigenic properties of the viral surface glycoproteins hemagglutinin (HA) and neuraminidase (NA). So far, 16 HA and 9 NA subtypes have been identified in various organisms. Furthermore, based on their virulence for poultry, influenza A viruses are classified as low pathogenic avian influenza (LPAI) or highly pathogenic avian influenza (HPAI). Among the various LPAI subtypes, it is primarily the hemagglutinin subtypes H5 and H7 that have the potential to switch from LPAI to HPAI. HPAI variants have markedly increased virulence in domesticated and wild birds and are occasionally associated with an ability to spread beyond species barriers to humans, horses, swine, and other mammals [1], [2]. The transformation from low to highly pathogenic virus is associated with an introduction of basic amino acid residues in the cleavage site of the HA protein [3]. Because of the ability of LPAI H7 to become a HPAI, which is a threat to the poultry species and to humans [4], LP H7 virus from Swedish mallards has been chosen for the development of vaccines to prevent and/or control infections in poultry and humans. In the recent years, many subunit vaccines have been developed against various HPAI H5 and H7 strains using HA of LPAI by traditional reverse genetics approaches in embryonated chicken eggs [5], [6], [7]. At the moment, traditional vaccines produced in eggs with inactivated whole-virus are combined with the Differentiating Infected from Vaccinated Animals (DIVA) system, but this approach have several limitations as the need for a large supply of specific pathogen-free (SPF) embryonated eggs, long production times, risk of introduction of point mutations during propagation (antigenic drift) and safety concerns for the production workers. As a result, many cell culture-based approaches have been investigated to develop vaccines, *i.e*. Madin Darby Canine Kidney (MDCK) cells [8], Vero (African green monkey) cells [9], PerC.6 cells [10], HEK-293 cells [11] and baculovirus expression vector system in insect cells [12].

Recently, good progress has been made in the production of plant-based antigens to overcome the problems associated with existing vaccine production systems. Plant-based expression system offers several advantages in terms of low capital investment, time efficiency, high yield, and a lower risk of contamination with human pathogens. Successful expression of plant-based vaccine antigens or virus-like particles have been established against various infectious microorganisms including enterotoxigenic *Escherichia coli* Lt-B antigen [13], *Bacillus anthracis* protective antigen [14], hepatitis B surface antigen [15], foot and mouth disease virus structural protein VP1 [16], rabies virus [17], cholera toxin B subunit [18] and influenza virus [19], [20]. Since 2008, many vaccine antigens for various subtypes and strains of influenza A virus have been expressed transiently in plants, including human H1N1 [21], human H3N2 [22], [23] and avian H5N1 [21], [24], [25], [26], [27].

The HA protein is a key antigen for generating protective immunity in response to influenza virus [28]. Also, recombinant HA antigens produced in plants have been shown to be immunogenic and safe in various animal models [22], [24], [27]. In 2006, Dow AgroSciences developed a plant-based poultry vaccine for Newcastle disease virus using a tobacco cell culture production system [29]. The present study was undertaken to investigate the possibility of producing biologically active recombinant full-length hemagglutinin (rHA0) of an H7N7 LPAI isolated from a wild Swedish mallard ducks in *Nicotiana benthamiana* as a subunit vaccine candidate against influenza in poultry and humans.

## Materials and Methods

### Viruses, bacterial strains, plant material and growth conditions

In this study, we used a characterized H7 influenza A virus from a mallard duck *Anas platyrhynchos* as the basis for all further experiments. This virus was originally collected in 2004 as part of an ongoing AIV surveillance at the Ottenby Bird Observatory on Öland, a Swedish island in the Baltic Sea [30], [31], and influenza A virus was detected using a real-time reverse transcriptase-polymerase chain reaction (rRT-PCR) assay based on the viral matrix gene [32]. For further characterization, the positive sample (A/mallard/Sweden/7206/2004(H7N7)) was inoculated in the allantoic cavities of 11-days old embryonated chicken eggs and the presence of virus in harvested fluids confirmed by hemagglutination assay using chicken erythrocytes (see section below).

*E. coli* (NovaBlue, Novagen) and *Agrobacterium tumefaciens* strain, LBA4404 [33] were used in molecular cloning experiments and were routinely cultured at 36±1°C and 25±1°C, respectively in Luria-Bertani (LB) media using appropriate antibiotics [34]. *N. benthamiana* plants were grown in a greenhouse and maintained at 23±2°C with a 16-h/8-h photoperiod.

### Isolation of RNA, RT-PCR and sequencing

Viral RNA was extracted from the strain of A/mallard/Sweden/7206/2004(H7N7) using a commercially available High Pure RNA Isolation Kit (Roche Diagnostics GmbH, Germany) according to the manufacturer's instructions. First strand cDNA was synthesized using 0.5 µg of Uni12 (M) (5′-AGCRAAAGCAGG-3′) primer [35] with RevertAid™ First Strand cDNA Synthesis Kit (Fermentas) as described by the manufacturer. Next, the *HA* gene was amplified using degenerate primers, HA F (5′-ATGAACACTCWAATCCTG-3′) and HA R (5′-TTATATACAARTAGTGCAC-3′). Cycling conditions were as follows: 95°C for 3 min followed by 35 cycles of 95°C for 1 min, 45°C for 1 min, 72°C for 4 min and a final elongation at 72°C for 10 min. PCR amplifications were performed using *Pfu* DNA polymerase (Fermentas). The gel purified product was cloned into pJET1.2 vector and transformed into competent *E. coli* NovaBlue cells according to the manufacturer's instructions. The identity of the cloned gene was verified by sequencing.

### Phylogenetic analysis and prediction of localization

The H7 amino acid sequence derived from the nucleotide sequence (HA) of the A/mallard/Sweden/7206/2004(H7N7) deposited in GenBank was aligned together with H7 sequences retrieved from public databases using the MAFFT program [36], [37] implemented in the Geneious software. The ProtTest 3.0 program was used to determine the appropriate model of sequence evolution for viral H7 amino acid sequences [38]. Phylogenetic relationships between aligned sequences were inferred by using the Bayesian Markov-Chain Monte Carlo (MCMC) method integrated in the MrBayes 3.1 program [39]. In this program, the default settings were used except for the substitution model where the Jones model with gamma-distributed rate variation across sites was used [40], [41]. The MEGA 5.0 program [42] was used to visualize the resulting phylogenetic tree. Signal peptide cleavage site of HA was predicted using the SignalP prediction program [43].

### Plant expression vector construction

**Figure 1:**
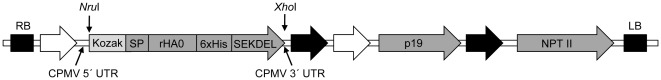
Schematic diagram of the pEAQ-*HT*-HA plant expression system construct used for agro-infiltration. The entire T-DNA region is shown (not to scale): Black boxes, T-DNA borders (RB, right border; LB, left border); white arrows, CaMV 35S promoters; grey arrows, open reading frames; black arrows, Nos terminators; and NPTII, neomycin phosphotransferase II gene.

The *HA* gene synthesis (GenBank accession no. AEP33185) was carried out by GeneArt AG, Germany (www.geneart.com) with codons optimized for the *Nicotiana tabacum* (codon adaptation index of 0.87) and with possible crucial sequence regions deleted including the internal TATA-boxes, chi-sites, ribosomal entry sites, sequence stretches with extreme GC or AT content, repetitive sequences, long hairpin loops, mRNA instability sequence motifs, RNA secondary structures, cryptic polyadenylation splicing sites, and transcription termination signals in order to maximize the rate of protein synthesis. The artificial gene was synthesized in the following context (Figure 1): *Nru*I restriction site, Kozak (GCCACC) a dicot preferred efficient initiation sequence of the translation in 5′-UTR of the transgene [44], amino-terminal native HA signal sequence (bp 1–54) replaced with signal sequence of tobacco *PR1a* for pathogenesis-related protein (90 bp) (GenBank accession no. X06930), removal of transmembrane domain (bp 1560–1632), sequences encoding the hexahistidine (6×His) tag for affinity purification, an endoplasmic reticulum (ER) retention signal peptide SEKDEL in the 3′-end to retain the truncated rHA0 in ER, duplicate stop codons to end the translation, and *Xho*I restriction site. The synthetic, codon optimized, recombinant *HA* gene was cloned into a Cowpea mosaic virus-based plant expression binary vector, pEAQ-*HT* [45], using the *Nru*I/*Xho*I restriction sites which resulted in the pEAQ-*HT*-HA construct; this recombinant plasmid was then transformed into competent *E. coli* NovaBlue cells by the heat shock method. Finally, all constructs were verified by sequencing.

### *Agrobacterium-* mediated transient expression in *N. benthamiana* leaves

The plant expression vectors pEAQ*-HT*-HA and pJL3:p19 [46], both expressing the post-transcriptional gene-silencing suppressor (P19) protein of Tomato bushy stunt virus (TBSV), were separately transformed into *A. tumefaciens* strain LBA4404 using the freeze-thaw method and confirmed by colony PCR. Resulting recombinant *Agrobacterium* strains were incubated in LB supplemented with 50 µg/mL kanamycin and 25 µg/mL rifampicin for 12–24 h with an optical density (OD_600_) of 1.2–1.4 and then pelleted by centrifugation at 2000 g for 20 min. The pellet was resuspended in infiltration buffer (10 mM 2-N-morpholino-ethanesulfonic acid (MES) pH 5.5, 10 mM MgCl_2_, and 100 µM acetosyringone). After 2 to 4 h incubations at room temperature to induce the virulent genes, *Agrobacterium* cultures carrying each expression vector were equally mixed and 5–6 full expanded mature leaves of 5–8 weeks old *N. benthamiana* plants were co-infiltrated using a 10 mL syringe without a needle. At 0, 3, 6, 9, 12 and 15 days post infiltration (dpi) (0 dpi represents non-infiltrated leaves), the leaves were harvested, frozen in liquid nitrogen and stored at −80°C until further use.

### Relative gene expression using quantitative real-time polymerase chain reaction

Recombinant gene expression in *Agrobacterium* leaves harvested at 0, 3, 6, 9, 12, and 15 dpi were measured by quantitative real-time polymerase chain reaction (qPCR) as described previously [47]. Briefly, RNA was isolated from the frozen plant tissue powder (100 mg) using Purelink™ Plant RNA Reagent kit (Invitrogen, Carlsbad, California, USA) following the manufacturer's instructions. The quality of RNA was determined by ethidium bromide-stained agarose gel electrophoresis and quantified using a Nanodrop ND-1000 spectrophotometer (Thermo Scientific). Genomic DNA contamination was removed by DNase I digestion (Fermentas, St Leo-Roth, Germany). First strand cDNA was synthesized as a 20 µL reaction, with 1 µg of total RNA as the template, 0.5 µg oligo(dT)_18_ primer and RevertAid™ H Minus-MuLV reverse transcriptase (200 U, Fermentas). Subsequently, the cDNA was treated with RNase H (Fermentas) to degrade remaining RNA and assayed by qPCR using HA specific primers (forward: 5′-GCGCTTTCATTGCTCCAGATAG-3′ and reverse: 5′- GCATCCACTTGAACCTCAGACT-3′) in a ABI Prism® 7500 Sequence Detection System (Applied Biosystems, USA). The qPCR reactions were conducted in a final volume of 20 µL using 1 µL of first strand cDNA, 10 µL Power SYBR® Green PCR master mix (Applied Biosystems) and 2 pmol of each forward and reverse primer. One-step qPCR reactions were performed under the following conditions: 50°C for 2 min, 95°C for 10 min followed by 40 cycles of 95°C for 15 s, 60°C for 1 min and dissociation stage at 95°C for 15 s, 60°C for 1 min, 95°C for 15 s. Target gene amplifications were verified during the melting curve analysis step. Amplification efficiencies of *HA*, *Ubi3* and *EF-1* genes were determined according to Pfaffl et al. [48]. Transcript levels of rHA0 were expressed as relative values normalized to the transcript level of Ubiquitin (*Ubi3*) (ubi3 forward 5′- GCCGACTACAACATCCAGAAGG-3′ and ubi3 reverse 5′-TGCAACACAGCGAGCTTAACC-3′) and elongation factor 1 α (*EF-1*) (EF-1 forward 5′-GATTGGTGGTATTGGAACTGTC-3′ and EF-1 reverse 5′- AGCTTCGTGGTGCATCTC-3′) genes, also measured by qPCR and used as an internal references [49]. Based on Cycle threshold (Ct) values from qPCR analyses, the expression of different genes evaluated by three technical replicates in two biological samples in the transcripts were quantified. Relative expression of the *rHA0* gene in relation to reference genes were calculated using the REST 2009 software V. 2.0.13 (Qiagen, Hilden, Germany) [48]. Relative values were calculated using the gene with lowest expression as a reference.

### Total soluble protein extraction and quantification

The total soluble protein (TSP) was extracted from the *Agrobacterium-*infiltrated *N. benthamiana* leaves as well as from non-infiltrated control leaves in liquid nitrogen with 1.5 w/v of extraction buffer containing 20 mM NaH_2_PO_4_, pH 7.5, 0.5 M NaCl, 10% (v/v) glycerol, 0.5% polyvinylpolypyrrolidone (PVPP) and 0.01% (v/v) plant protease inhibitor cocktail (Sigma, P9599) at 4°C. Inhibitors were added to the extraction buffer just prior to use. The protein concentration in extracts was determined by using the Bio-Rad protein assay using bovine serum albumin (BSA) as standard in spectrophotometer (ND-1000, Nanodrop®). SDS-PAGE and western blot analysis were used to determine expression efficiency of rHA0 at different expression times.

### Purification and quantification of plant-produced recombinant hemagglutinin

Extracts of TSP containing the soluble rHA0 expressed with a 6×His tag at the C-terminal were purified using immobilized metal ion adsorption chromatography (IMAC) by applying the plant extract on 5 mL HiTrap Chelating HP column (GE Healthcare) charged with Ni^2+^. After washing the column with binding buffer (20 mM sodium phosphate, pH 7.4, 0.5 M NaCl, 30 mM imidazole), bound protein was eluted (20 mM sodium phosphate, pH 7.4, 0.5 M NaCl and 500 mM imidazole) at 4°C. The concentration of rHA0 in eluted fractions was quantified (Bio-Rad protein assay) and the fractions having highest protein concentrations were pooled and desalted with a PD-10 column (GE Healthcare) equilibrated with phosphate-buffered saline (PBS) pH 7.4. The eluted fractions of purified enzyme were analyzed by SDS-PAGE, western blot and stored at −20°C until used. The experiment was performed three times with similar results.

### SDS-PAGE and western blotting

Forty micrograms of TSP from recombinant leaves and 5 µg of purified proteins were resolved on SDS–PAGE electrophoresis NuPAGE™ 4–12% Bis/Tris gels (Invitrogen) using NuPAGE™ 1× MES SDS Running Buffer (Invitrogen) according to manufacturer's instructions with a Novex™ X-Cell II™ mini horizontal gel electrophoresis unit. After electrophoresis, the gels were either stained with PageBlue™ Protein Staining Solution (Fermentas) following manufacturer's basic protocol or electro-transferred on to 0.45 µm polyvinylidene fluoride (PVDF) membranes (Millipore, Billerica, MA, USA) using a Novex™ X-Cell II™ Blot module as described by the manufacturer. PageRuler™ Prestained Protein Ladder (Fermentas) was included in SDS-PAGE and western blots as molecular weight markers. Immunodetection with Mouse Anti-His (Invitrogen) followed by incubation with Amersham ECL Anti-mouse IgG peroxidase-linked species specific antibody (GE Healthcare) was used to identify rHA0. The purified protein (5 µg) was also probed with polyclonal hemagglutinin (H7)-specific antiserum raised in rabbits against influenza strain, A/Eq/Praque/1/54 followed by incubation with Blotting Grade Goat Anti-Rabbit IgG (H+L) (Human IgG Adsorbed) Horseradish Peroxidase (HRP) (Bio-Rad, Hercules, USA). The rHA0 reacting with anti-His and anti-HA were visualized using Amersham ECL Western Blotting detection reagents (GE Healthcare) in accordance with manufacturer's procedures.

### Analysis of post-translational modifications

Protein N-glycosylation sites of rHA0 were predicted using the NetNGlyc 1.0 software available at URL http://www.cbs.dtu.dk/services/NetNGlyc/. Thereafter, the rHA0 glycosylation status was experimentally evaluated by an enzymatic reaction. Ten micrograms of purified N-glycosylated rHA0 were digested with peptide-N-glycosidase F as described by the manufacturer (PNGase F New England BioLabs, Beverly, MA, USA) to remove the N-linked glycans. The resulted N-deglycosylated protein (5 µg) was resolved by SDS-PAGE, then electro-transferred on to a PVDF membrane and analyzed by western blot as previously described. Control digests were carried out without PNGase F.

### Protein identification using LC-MS/MS

Purified rHA0 from SDS-PAGE gel was cut out, digested with trypsin and analyzed by liquid chromatography (LC) (Dionex, Breda, The Netherlands) coupled quadrupole mass spectrometry (MS/MS). Mass spectrometric data obtained from quadrupole Time of flight (Q-TOF) Ultima instrument (Waters, Sollentuna, Sweden) were recorded with charge state of 2 and 3. For product identification, tandem MS spectra of the tryptic peptides were analyzed using the Mascot Daemon (version 2.3; Matrix Science Ltd, London, UK) database with settings for carbamidomethylation of cysteines fixed and variable oxidation of methionine residues, respectively.

### Hemagglutination and hemagglutination inhibition (HI) assay

Biological activity of plant-produced rHA0 was evaluated by hemagglutination assay [50]. Triplicates of purified rHA0 were serially two-fold diluted in PBS (pH 7.4) and incubated at 4°C for 2 h with 25 µl of 1% chicken erythrocytes in U bottom 96-well microtiter plates. Bovine serum albumin (Sigma) was used as a negative control. The hemagglutination titer (HT) was determined in wells with lowest hemagglutinin concentration (µg/mL) causing complete hemagglutination.

Hemagglutination inhibition (HI) assays were performed to evaluate antigenic properties of rHA0 by using subtype-specific hyperimmune rabbit antiserum raised against virus isolates of the most common H1–12 subtypes (Table 1), as previously described [50], [51]. Briefly, rHA0 was diluted in PBS to a protein concentration corresponding to 4 hemagglutination units (HAU) and tested with hyperimmune antisera diluted 1∶80, 1∶160, 1∶320 and 1∶640, respectively for their ability to inhibit agglutination of chicken erythrocytes. For this, following 30 min incubation of virus and subtype-specific antisera at 30°C, 25 µl of a 1% chicken erythrocyte suspension was added and then incubated for an additional 1 h at 4°C. In this assay, the Swedish H7 isolate was used as positive control, and hyperimmune rabbit antisera diluted in PBS without protein antigen (to check non-specific agglutinination) was used as negative control.

**Table 1:** Rabbit antisera raised against influenza A virus subtypes H1-H12.

Sera	HA subtype	Anti-rabbit
1	H1a	A/sw/Shope/56
2	H1b	A/duck/Alberta/35/76
3	H2	A/Singapore/1/57
4	H3a	A/Eq/Miami/1/63
5	H3b	A/duck/Ukraine/63
6	H4	A/duck/Czech/1/56
7	H5	A/duck/Hongkong/205/77
8	H6a	A/Ty/Mass/1/65
9	H6b	A/Shearwater/Aus/1/72
10	H7	A/Eq/Praque/1/54
11	H8	A/Ty/Ontario/6118/68
12	H9	A/Ty/Wisconsin/1/66
13	H10	A/Ck/Germany/N/49
14	H11a	A/Dk/England/56
15	H11b	A/Dk/Memphis/546/74
16	H12	A/Dk/Alberta/60/76

## Results

### Virus characterization

The isolated influenza virus A/mallard/Sweden/7206/2004(H7N7) subtype was determined by serological methods and confirmed by sequencing based on nucleotide sequences of hemagglutinin (GenBank accession no. JN674638) and neuraminidase (GenBank accession no. JN674639).

**Figure 2:**
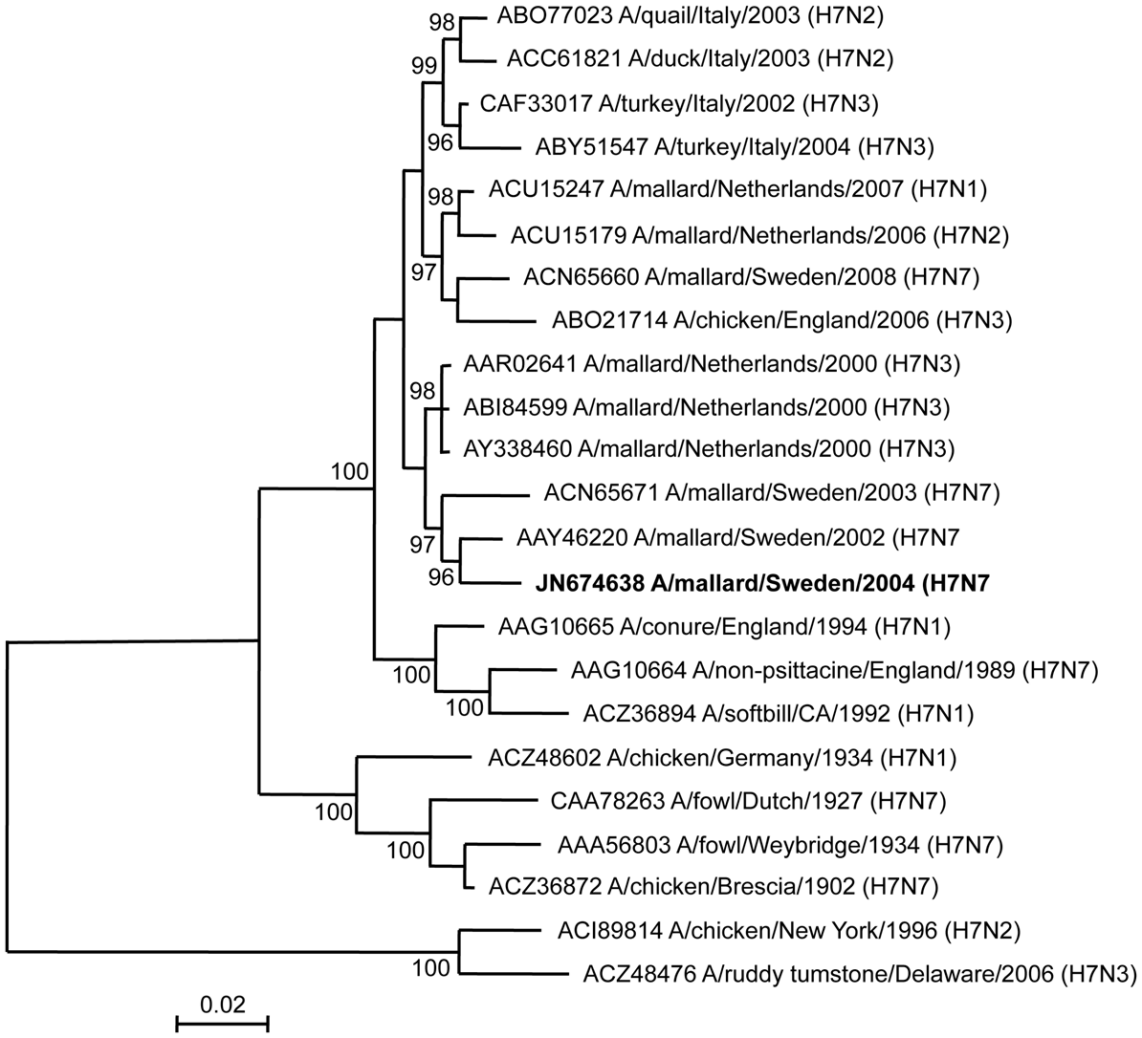
Phylogenetic relationships between the A/mallard/Sweden/7206/2004(H7N7) isolate and representative H7 sequences available in public databases. Bayesian phylogeny of HA amino acid sequences (denoted by GenBank accession number and isolated description) was inferred using the MrBayes 3.1.2 program and the Jones model of substation with gamma-distributed rate variation across sites. Nodes with posterior probability support ≥95% are indicated. The Swedish H7 sequence isolated is highlighted in bold face. Scale bar indicates the number of amino acid changes per branch length.

In order to compare phylogenetic relationships of the H7 sequence with temporally and spatially diverse H7 sequences isolated from different bird species, a 1683-bp fragment of the *HA* gene encoding 561 amino acids site was obtained by RT-PCR, cloned and sequenced. After aligning obtained amino acid sequence with H7 sequences from databases, and refining initial phylogenetic trees containing large number of sequences, 20 European and 2 North American H7 sequences, representing clades in the initial trees were retained in the final tree. This inferred Bayesian tree revealed two major genetic lineages representing European avian and North American avian influenza isolates, respectively (Figure 2). The European avian genetic lineage further branched into two temporal sublineages corresponding to the periods, 1902–1934 and 1989–2008. Within the later of these clades, our strain A/mallard/Sweden/7206/2004(H7N7) was most closely related with other North European isolates, isolated from Swedish and Dutch mallards from 2000 to 2003.

### Transient expression, purification and quantification of recombinant hemagglutinin

**Figure 3:**
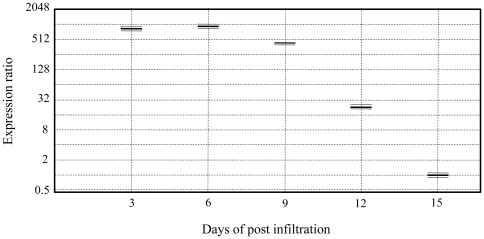
Relative expression of plant-produced recombinant hemagglutinin was measured by quantitative real-time PCR. The expression levels was measured at 3, 6, 9, 12 and 15 dpi (days post infiltration) and are normalized to expression of control genes, the ubiquitin (*ubi3*) and elongation factor-1 (*EF-1*). The results represent two separate experiments, each time performed including three technical replicates.

After agro-infiltration of *N. benthamiana* leaves with the plasmids, pEAQ-*HT*-HA containing codon optimized, artificially synthesized gene (*rHA0*) encoding 565 amino acid protein and *Agrobacterium* strain harboring the *p19* (pJL3:p19) gene, a suppressor of gene silencing to enhance *rHA0* gene expression, the qPCR analysis was carried out to check the expression pattern of rHA0 at different time points. In order to verify *rHA0* gene expression and determine the relative transcript levels of genes until 15 dpi, we harvested the infiltrated *N. benthamiana* leaves at 0, 3, 6, 9, 12 and 15 dpi and measured mRNA levels by qPCR analysis. To ensure the comparability of transcripts at different time points, all qPCR reactions were performed with equal quantity of total RNA and amplification efficiency was ensured by standard curve tests measuring ten-fold serial dilutions of 1 µg of cDNA from control and infected leaf samples. Amplification efficiencies of *rHA0* and the reference genes, *ubi3* and *EF-1* were found to be 83%, 86% and 91%, respectively, indicating relevant amplification and accurate quantification of recombinant transcripts in the qPCR analyses. Homogeneity and specificity of amplified products were confirmed by melting curve analysis (data not shown). Relative gene expression was determined comparing the Ct value of transcripts at different time points (3, 6, 9, 12 and 15 dpi). The results showed the significant level of the *rHA0* gene was transcribed through-out tested time period, but also that expression levels varied considerably, ranging from 17.9 to 29.2. The relative rHA0 expression peaked 6 dpi, remained relatively stable until 9 dpi, after which (*i.e*., at 12 and 15 dpi) expression reduced down considerably (Figure 3). A quantitative estimate of the rHA0 activity showed that transcription was approximately 1000-fold higher at 6 dpi compared to the number of mRNA copies at 15 dpi. Transcripts of rHA0 were not detected in the negative control (0 dpi).

**Figure 4:**
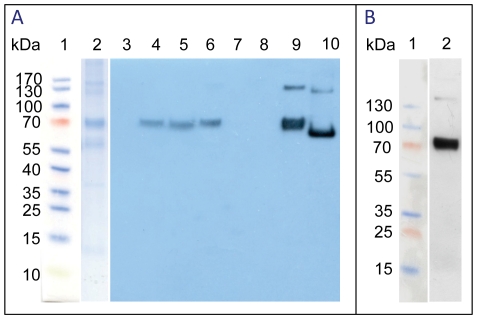
Plant-produced recombinant full-length hemagglutinin (rHA0) analysis using SDS-PAGE and western blot. A: Probe with anti-His antibodies, Lane 1, molecular weight marker; lane 2, purified rHA0 (5 µg) from total soluble protein (TSP) at 6 days post infiltration (dpi) (SDS-PAGE); lane 3, TSP (40 µg) from untreated *Nicotiana benthamiana* leaves (negative control; 0 dpi); lanes 4 to 8, TSP (40 µg protein per lane) from the agro-infiltrated leaves of *N. benthamiana* at 3, 6, 9, 12 and 15 dpi; lane 9, purified rHA0 (5 µg) at 6 dpi; lane 10, purified rHA0 (5 µg) at 6 dpi after PNGase F digestion. The shift to a lower molecular mass upon treatment with PNGase F is indicative of N-linked glycosylation on rHA0; B: Probe with anti-HA antibody, Lane 1, molecular weight marker, lane 2, purified rHA0 (5 µg) at 6 dpi.

After confirming activity of the *rHA0* gene, the TSP was probed for viral HA protein. The fraction of soluble proteins, extracted from recombinant plants at 6 dpi was purified by IMAC using Ni^2+^. Purified plant-produced rHA0 protein at a yield of 210 mg/kg fresh weight of leaves was eluted from the affinity column, and expression level was estimated to be 9.7% of TSP. To ensure the rHA0 transcripts correlated with expression of protein, we analyzed fractions eluted from affinity column by SDS-PAGE and western blot analyses. SDS-PAGE and western blot analysis of transiently expressed rHA0 from the TSP of infiltrated leaves at 0, 3, 6, 9, 12 and 15 dpi and purified protein confirmed the expression of rHA0 at the size of ∼70 kDa (Figure 4A and 4B) that is specifically recognized by mouse anti-His and rabbit anti-HA antibodies. Probing with the anti-HA antibodies showed that rHA0 is synthesized and purified as a monomeric polypeptide and that it is not proteolytically cleaved into HA1 and HA2, as it is during viral infection (Figure 4B, lane 2). The expressed rHA0 was about 10 kDa larger than the predicted native protein size after cleaving the signal peptide because of restriction site of *Xma*I, 6×His tag at the C-terminus end and possibly unknown post-translational modifications. Based on western blot results, it is therefore proposed that rHA0 were expressed until 9 dpi and strongly expressed at 6 dpi in accordance with qPCR results. The experiments were repeated three times and the results were reproducible.

Sequence analysis of rHA0 predictions suggested the existence of five N-linked glycosylation sites in the viral protein. The glycosylation of rHA0 was confirmed by digestion of purified protein with PNGase F followed by SDS-PAGE/western blot analysis. On the western blot membrane, the PNGase F activity was visualized as a shift in molecular mass, changing from ∼70 kDa for the uncleaved rHA0 protein to 60 kDa for the enzymatic cleavage product (Figure 4A); this indicates that the rHA0 produced in *N. benthamiana* is post-translationally modified by the addition of N-linked glycans. Authenticity of rHA0 was further confirmed by the analysis of purified protein with LC-MS/MS covering 25 per cent amino acid sequences (data not shown).

### Functional characterization of recombinant hemagglutinin

**Figure 5:**
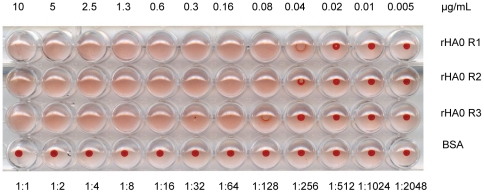
Hemagglutination assay. Triplicates of purified plant-produced hemagglutinin (rHA0) were two-fold serially diluted beginning with a concentration of 10 µg/mL from the stock of 400 µg/mL and mixed with chicken erythrocytes. Wells in the bottom row contains bovine serum albumin (BSA) as a negative control starting with a concentration of 10 µg/mL from 400 µg/mL stock. The lowest concentration (µg/mL) of the well showing complete agglutinating activity of erythrocytes was considered as hemagglutination titers (HT).

The activity of purified plant-produced rHA0 was determined by hemagglutination. The hemagglutination of chicken erythrocytes in the U-well micro-titer plate with plant-produced rHA0 was visually observed in wells with protein concentration higher than 0.12 µg/mL. No hemagglutination activity was observed in wells with rHA0 concentration lower than 0.06 µg or in negative controls containing BSA (Figure 5).

**Figure 6:**
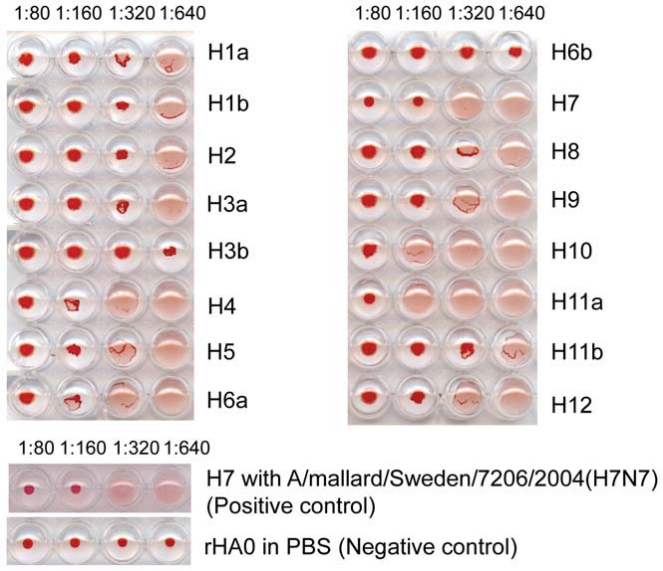
Hemagglutination inhibition assay. For the assay, 4 HAU of purified plant-produced recombinant hemagglutinin (rHA0) were mixed with 2-fold serial diluted hyperimmune rabbit antiserum (starting at a dilution of 1∶80) generated against various subtypes of viral strains or phosphate buffer saline (PBS) (negative control). Native Swedish H7N7 influenza A virus isolate was used as positive control. Formation of immune complexes was allowed for 30 min at 37°C, before incubation with chicken erythrocytes for 1 h at 4°C.

To examine antigenic properties of plant-produced rHA0, HI assay with 0.3 µg/mL (corresponding to 1 HAU) of purified protein was analyzed and compared to HI results obtained with 1 HAU of native H7N7 virus. The analysis showed that hyperimmune rabbit antiserum raised against H7 subtype virus (A/Eq/Praque/1/54) blocked rHA0 induced hemagglutinin activity at a dilution titer of 1∶160, a result corresponding to that observed with native virus (Figure 6). No HI activity or no serologic cross-reactivity was displayed with hyperimmune rabbit antiserum generated against the different subtypes (H1–6 and H8–12) of AIV (Figure 6). The whole experiment was repeated on three occasions with similar results.

## Discussion

The objective of our study was to explore the transient expression of HA antigens from the isolated LPAI of wild Swedish mallard ducks in *N. benthamiana* amenable for the development of plant-produced vaccines.

LPAI infections in mallards do not produce overt disease, but is associated with weight loss in free-flying individuals [31]. In poultry, however, LPAI infection can cause mild respiratory disease, depression and reduced egg production in laying turkeys and chickens. AIV H7 outbreaks in poultry have been reported from various countries after initial transmission from wild birds and subsequent adaptation of LPAI in poultry species [52], [53], [54], [55]. In 2003, an outbreak of HPAI H7N7 closely related to a LPAI H7N7 virus isolated from mallards, infected 89 people and caused an acute fatal respiratory illness syndrome of an attended veterinarian [2]. Hence, LPAI H7 infection is a great concern due to its high potential to become highly virulent in poultry and the possibility of cross-species transmission to humans [2], [5], [6].

The phylogenetic analysis of HA sequence from the strain, A/mallard/Sweden/7206/2004(H7N7) revealed a close relation to North European LPAI isolates, and particularly to other Swedish H7 virus sequences. In the obtained HA sequence, a catalytic cleavage site typical of H7 LPAI, with only basic amino acids at positions −1 (arginine) and −3 (lysine) was observed [52], [56]. Several researchers have suggested that HPAI in poultry have evolved from LPAI, and that this virus after adaptation in populations of domesticated birds developed the pathogenic traits [4], [52], [57].

In recent decades, a large number of studies have been initiated to investigate the possibility to express recombinant vaccine antigens in plants as these antigens are considered as safe, low cost, easy to produce, rapid to upscale, and less vulnerable to contamination with animal pathogens compared with traditional inactivated or live attenuated egg-based vaccines. For example, immunogenicity associated to administration of H5 antigens produced in plants has been reported [13], [14], [15], [16], [24], [26]. HA is the major glycoprotein involved in attachment of the influenza virus to sialic acid-containing host cell receptors, and as a surface protein in the native viral particle, this protein is immunogenic [58]. In this study, the potential of using *N. benthamiana* for production of influenza A virus HA surface antigen was investigated. For transgenic protein expression, the HA gene of the AIV subtype H7 was expressed as soluble recombinant 6×His tagged fusion protein in the Cowpea mosaic virus- based transient plant expression system. To achieve high expression levels of rHA0 in *N. benthamiana*, multiple criteria were considered in the construction of plant expression vector. Previous studies suggested that incorporating Kozak sequence in the upstream of the start codon can significantly increase the efficiency of translation in eukaryotic cells [44], [59]. Therefore, a Kozak (GCCACC) plant translation initiation sequence was included in front of the start codon of rHA0 to ensure high expression levels. In addition, it has been reported that HA is an N-linked glycoprotein which is directed into the secretory pathway and retained in the ER for post-translational modifications [24], [26]. For the efficient production of H7 protein in plant cells, which is normally expressed in epithelial cells of infected birds, the HA was modified with a native signal peptide sequence by introducing a cleavable apoplast targeting N-terminal signal sequence from tobacco pathogenesis-related protein PR1a, full-length HA coding sequence containing both the HA1 and HA2 domains, and C-terminal 6×His tag to facilitate purification [22]. Furthermore, several researchers included an ER retention signal, SEKDEL in the C-terminal as it is expected to sequester the protein in the ER to fold correctly, as well as to increase protein stability and preventing the entry of protein into the Golgi apparatus, the site for plant specific glycosylation [22], [23]. The ER retention signal was followed by two stop codons to stop the translation efficiently and also deleted the transmembrane domain of HA [22], [23], [26]. Also, previous reports suggested that the synthetic codon adapted gene to tobacco with optimized GC content while removing sequence repeats, cryptic splice sites and RNA destabilizing sequence elements expression in tobacco resulted in the high level accumulation of recombinant functional proteins [60], [61]. Therefore, we synthesized HA artificially following above mentioned parameters to achieve the high level expression in *N. benthamiana.*

Previously, it has been shown that transient *Agrobacterium*-mediated transformation with Tobacco mosaic virus (TMV) based viral vectors produces vaccine antigens in tobacco [24], [26]. In this study, we expressed the HA glycoprotein of H7N7 LPAI in *N. benthamiana* with transient *Agrobacterium-*mediated leaf infiltration using a CPMV-based vector. The relative expression increase of H7 AIV *i.e*. rHA0 at 6 dpi (∼1000-fold) in the infiltrated leaves, was measured by qPCR assays. The results are consistent with previous findings showing that upon infection of *N. benthamiana* with a TMV-based vector system, an increase of transgenic protein is observed at 6 dpi [62].

Plants expressing CPMV proteins, rHA0 as well as p19 protein, started to show symptoms of stunting of shoot growth and wilting of leaves at 9 dpi after agro-infiltration. The process continued until complete necrosis was observed at 18 dpi. Possibly, the viral proteins exert a toxic effect in plant cells by an unknown mechanism. However, it has also been suggested that the expression of p19 protein may induced a systemic necrosis in *N. benthamiana* [63], [64]. Also, Lacorte et al. [65] observed necrotic reactions while expressing genes from chicken anemia virus using TMV and Potato virus X based vectors in *N. benthamiana*.

Recent studies of *N. benthamiana*-produced purified HA vaccine antigens reported production level of 20 to 100 mg/kg FW [22], [24]. In our study, transiently expressed purified rHA0 fusion protein attained expression levels of 210 mg/kg FW, which are approximately 9.7% of TSP in *N. benthamiana*.

In a western blot analysis of expressed proteins in reducing conditions using a His tag specific monoclonal antibody, a single ∼70 kDa band was detected, a molecular weight of approximately 10 kDa higher than expected molecular weight. These results are in agreement with a previous report showing that HPAI H5N1 hemagglutinin expressed at ca. 70 kDa in *N. benthamiana* [24]. Also, the analysis of the HA sequence using NetNGlyc 1.0 program predicted five N-glycosylation sites in rHA0, which would have contributed to the increased molecular weight. Predicted post-translational glycosylation of rHA0 was confirmed by enzymatic deglycosylation cleavage with the PNGase F enzyme and subsequent visualization of a 60 kDa band on the western blot membrane. These results indicate that rHA0 expressed in *N. benthamiana* are modified with glycans in a way similar to HA proteins produced in bird's cells during virus infection. Also, these observations confirm that rHA0 enters into ER secretory pathway of the plant and that it is extensively expressed in subcellular organs.

In the present study, we demonstrated that the rHA0 antigen was biologically active by confirming its intrinsic hemagglutination activity, observed at concentrations as low as 0.12 mg/mL. Furthermore, purified rHA0 epitopes were detected by hyperimmune rabbit antissera raised against H7 AIV and hemagglutinin inhibition titres of rHA0 exhibited similar titres to those detected in corresponding analysis with native H7 AIV isolate (*i.e* A/mallard/Sweden/7206/2004(H7N7)). These results indicate that a plant-produced H7 antigen is equivalent to the virus antigens in the context of immune recognition, and possibly that rHA0 retained its native structure and biological activity after purification. Results from HI assay also indicate that rHA0 interacts with other subtype specific antisera in a non-specific way seen as indistinct diffused buttons with uneven edges over the bottom of the wells. Combined results of hemagglutination and HI demonstrate that the expression of rHA0 in *N. benthamiana* is correctly translated, properly glycosylated, folded, fully functional and antigenically specific to H7.

To our knowledge, this is one of the more promising approaches, examining the possibility of generating viral vaccine antigens from LPAI H7 in a plant-based expression system. Obtained results, including high level expression of biologically active influenza A virus HA antigen, suggest that plants may be an important complement to traditional vaccine production methods, and thereby making vaccine more accessible worldwide.
